# Mannose-Binding Lectin Levels and Carotid Intima-Media Thickness in Type 2 Diabetic Patients

**DOI:** 10.1155/2016/8132925

**Published:** 2015-11-10

**Authors:** Miklós Káplár, Shah Sweni, Julianna Kulcsár, Barbara Cogoi, Regina Esze, Sándor Somodi, Mária Papp, László Oláh, Mária Tünde Magyar, Katalin Szabó, Katalin Réka Czuriga-Kovács, Jolán Hársfalvi, György Paragh

**Affiliations:** ^1^Division of Metabolic Diseases, Department of Internal Medicine, Faculty of Medicine, University of Debrecen, Nagyerdei Körút 98, Debrecen 4032, Hungary; ^2^Royal London Hospital, Barts Health NHS Trust, Whitechapel Road, London E1 1BB, UK; ^3^Division of Gastroenterology, Department of Internal Medicine, Faculty of Medicine, University of Debrecen, Nagyerdei Körút 98, Debrecen 4032, Hungary; ^4^Department of Neurology, Faculty of Medicine, University of Debrecen, Móricz Zsigmond Körtér 22, Debrecen 4032, Hungary; ^5^Clinical Research Center, Faculty of Medicine, University of Debrecen, Tuzoltó Utca 37-47, Budapest 1094, Hungary; ^6^Department of Biophysics and Radiation Biology, Semmelweis University, Budapest, Hungary

## Abstract

*Introduction*. Mannose-binding lectin (MBL) activates complement system and has been suggested to play a role in vascular complications in diabetics. Carotid intima-media thickness (cIMT) detects subclinical atherosclerosis. We evaluated the association of MBL and IMT in type 2 diabetic (T2DM) patients.* Methods*. Serum MBL levels and cIMT were measured in a total of 103 diabetics and in 98 age-matched healthy controls.* Results*. There was no significant difference in MBL level in T2DM versus controls. As expected, IMT was significantly higher in T2DM patients than in controls (*P* = 0.001). In T2DM, the lowest cIMT was seen in patients with normal MBL level (500–1000) while cIMT continuously increased with both high MBL and absolute MBL deficiency states. This was especially significant in high MBL versus normal MBL T2DM patients (*P* = 0.002). According to multiple regression analysis the main predictors of IMT in T2DM are age (*P* < 0.003), ApoA level (*P* = 0.023), and the MBL (*P* = 0.036).* Conclusions*. Our results suggest a dual role of MBL as a risk factor for cIMT in T2DM. MBL may also be used as a marker of macrovascular disease, as both low and high levels indicate the susceptibility for atherosclerosis in T2DM.

## 1. Introduction

Diabetes mellitus is the most prevalent and independent risk factor for atherosclerosis/cardiovascular diseases (CVD) [1] including coronary artery disease (CAD). CVD is the leading cause of mortality in type 2 diabetics (T2DM) [1]. Several inflammatory markers (fibrinogen, CRP, IL-18, and TNF-alpha) have been associated with markers of asymptomatic atherosclerosis in type 2 diabetics [2]. Mannose-binding lectin (MBL) has been suggested to play a role in the pathogenesis of CVD in diabetics [3, 4].

MBL, an important member of innate immunity, activating the lectin pathway of complement, is a weak-acute phase reactant [5] and its level increases only two- to threefold temporarily in response to different stress factors [6].

Previous studies analyzing the role of MBL in CVD have demonstrated a proatherogenic as well as an antiatherogenic role. Low MBL pheno- or genotype has been associated with higher risk of atherosclerosis [7, 8], arterial thrombosis [9], coronary artery disease [10, 11], bypass graft occlusion [12], and carotid artery plaques [13]. However, high MBL levels or normal genotypes have also been associated with coronary artery disease [14] and restenosis after carotid endarterectomy [15]. Furthermore, in type 2 diabetic population, low MBL genotype is associated with increased risk of cardiovascular events [16] and poorer outcomes of myocardial infarction [17]. Whereas high MBL is associated with increased mortality [18], disease progression, and nephropathy [19], protective role of high MBL in diabetics has also been suggested [20].

B mode ultrasonography mediated measurement of carotid artery intima-media thickness (cIMT) permits the study of subclinical carotid atherosclerosis, and patients at high risk for CVD can be identified [21]. Studies assessing correlation of MBL pheno- or genotypes with carotid intima-media thickness [22, 23] are conflicting. Independent of traditional risk factors, a quadratic U-shaped relation between serum MBL and cIMT was demonstrated in patients with rheumatoid arthritis. It supported a notion; both high and low MBL may play a role in CVD [24].

The objective of this study was to investigate hypothetical dual role of serum MBL level in intima-media thickness development among type 2 diabetic patients.

## 2. Methods

### 2.1. Study Design

After obtaining an institutional ethical clearance and an informed consent from participants, type 2 diabetic patients (T2DM) attending diabetes outpatient clinic at the Department of Internal Medicine, University of Debrecen, were recruited for the study. Amongst the diabetic patients, patients with severe hypoglycemia, hyperglycemia, diabetic ketoacidosis within three months prior to sample collection, active infections, malignancy, and other comorbid illnesses were excluded. We excluded pregnant females from our study. Only Caucasians aged greater than/equal to 24 and less than/equal to 78 years were included. Healthy age-matched subjects without diabetes were recruited as controls. Subjects with smoking pack years ≥ 20 were considered as smokers. Criteria for hypertension included patients with three consecutive arterial blood pressure values equal to or exceeding 140/90 mmHg or use of antihypertensive medication. Hypertensives (cases and controls) were included only if they were well treated and did not have hypertensive crisis within three months prior to sample collection. Of the subjects who met our inclusion criteria, a total 103 T2DM patients and 98 controls matched for age were involved in the study. An informed consent, detailed history and sociodemographic details, were obtained from all participants. Laboratory analysis and measurement of IMT was carried out by personnel blinded to clinical status of the subjects.

### 2.2. Laboratory Analysis

The blood samples were taken after overnight fasting of at least 10 hours by qualified staff. Fresh serum samples were used to analyze serum glucose, HbA1c, and lipid profile using standard methods at the Department of Laboratory Medicine, University of Debrecen. High sensitivity C-reactive protein (CRP) was measured using Integra 700 Auto Analyzer system (Roche, Basel, Switzerland). Serum samples were stored at −80° Celsius, for further analysis of MBL levels. These samples were defrosted just before analysis of MBL.

### 2.3. MBL Levels

MBL concentrations were measured by sandwich enzyme-linked immunosorbent assay as described and used previously at our institution [25]. In short, murine monoclonal antibody (Statens Serum Institut, Denmark), which preferentially detects functional MBL oligomers, was used. Microtiter plates (flat bottom, high binding capacity, Greiner Bio-One, Mosonmagyarovar, Hungary) were coated with primary antibody (HYB131-01) at 10 *μ*L/10 mL, that is, 1/1000 dilution in Tris-buffered solution (TBS), and were incubated overnight at 4° Celsius. Sera diluted 5-fold, 25-fold, and 125-fold in dilution buffer and serial twofold dilutions of an MBL serum standard (BioPorto Diagnostics A/S) were added to the wells and incubated at 37° Celsius for 90 minutes in a wet chamber. Biotin-labeled secondary (HYB131-01B) antibody at 1.25 *μ*L/10 mL (1/8000 dilution) in TBS, 0.05% Tween-20, and 0.25 *μ*M EDTA (TBS-T-EDTA) was incubated at room temperature for 90 minutes in a wet chamber. Horseradish peroxidase-conjugated biotin-avidin complex (Vectastain, Vector Laboratories Inc., Burlingame, CA) and substrate solution containing tetramethylbenzidine dihydrochloride (TMB, Sigma Aldrich, Schnelldorf, Germany) were used for obtaining color reaction. Reaction was stopped with 2 M H_2_SO_4_. Absorbance was measured at 450 nm using an ELISA reader Infinity 200 M (Tecan Austria GmbH, Grödig Austria). MBL concentration was estimated by reference to the serum standard. The results were calculated by the Magellan software program of the ELISA reader, using Marquardt curve fitting.

### 2.4. Carotid Intima-Media Thickness (cIMT) Measurement

Philips HD 11 XE ultrasound equipment with a 7.5 MHz linear transducer was used to measure IMT (mm). Online measurements of IMT were performed in the far artery wall of the common carotid arteries, 10 mm proximal to the carotid bulb [21]. All measurements were performed on frozen, enlarged images at end-diastole, and the transducer was in the mediolateral direction. IMT was measured on a 1 cm segment. In each of these 1 cm segments, 10 measurements of IMT were performed at 1-mm increments on both sides. The mean IMT of the 20 values in each patient was calculated.

### 2.5. Statistical Analysis

All the statistical calculations were performed using IBM SPSS Statistics 20.0 program package. MBL, serum triglyceride (Tg), HDL cholesterol, and CRP showed a nonnormal distribution; hence, median and interquartile ranges are used. All other values are given as means ± standard deviation (SD). Spearman correlation analysis was used to correlate IMT with MBL levels in different groups of patients. Furthermore, because both the MBL deficiency and the high MBL are associated with elevated cardiovascular risk, each group (T2DM and control) was subdivided according to serum MBL levels into 4 subgroups: absolute MBL deficiency (<100 ng/mL), intermediate MBL deficiency (100–500 ng/mL), normal MBL (500–1000 ng/mL), and high MBL levels (>1000 ng/mL) to analyze the possible nonlinear association between MBL and IMT. IMT in different subgroups were compared using one-way analysis of variance (ANOVA). We used binary logistic regression with dichotomous categorization of cIMT for high (>0.7) and low (<0.7) IMT groups. We also used multiple linear regression analysis to evaluate the multivariate associations between IMT (dependent variable) and CVD risk factors (predictors).

## 3. Results

### 3.1. Baseline Characteristics and MBL

**Figure 1:**
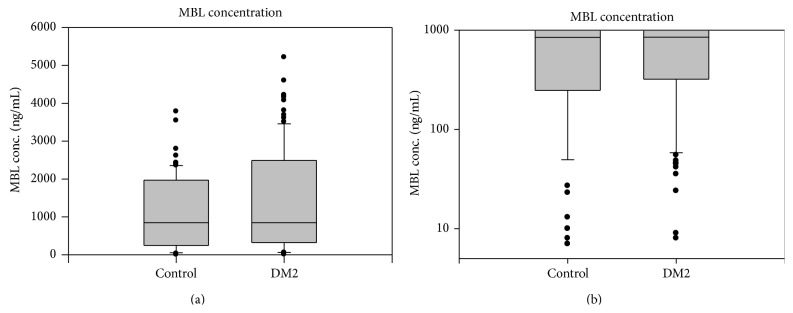
Box and Whisker plots of Mannose-binding lectin (MBL) distribution in type 2 diabetics and controls. The median (lower and upper quartiles) levels for MBL in T2DM and controls were 819 ng/mL (321–1871) and 846 ng/mL (247–1969), respectively. There was no significant difference between either group.

Baseline characteristics of diabetics and controls are summarized in Table 1. Both groups (T2DM and controls) were matched for age. Moreover, they did not significantly differ in gender and number of smokers. Although the number of patients with hypertension in T2DM group was significantly higher but all of them were well treated and had normal blood pressure. The median (lower and upper quartiles) levels for MBL in T2DM and controls were 819 ng/mL (321–1871) and 846 (247–1969), respectively. There was no significant difference between either group (Figure 1). Through Spearman correlation analysis, MBL was not associated with age, gender, serum glucose, HbA1c, hypertension, CRP, and total cholesterol in both diabetic and control groups and with duration of diabetes in the T2DM group.

**Figure 2:**
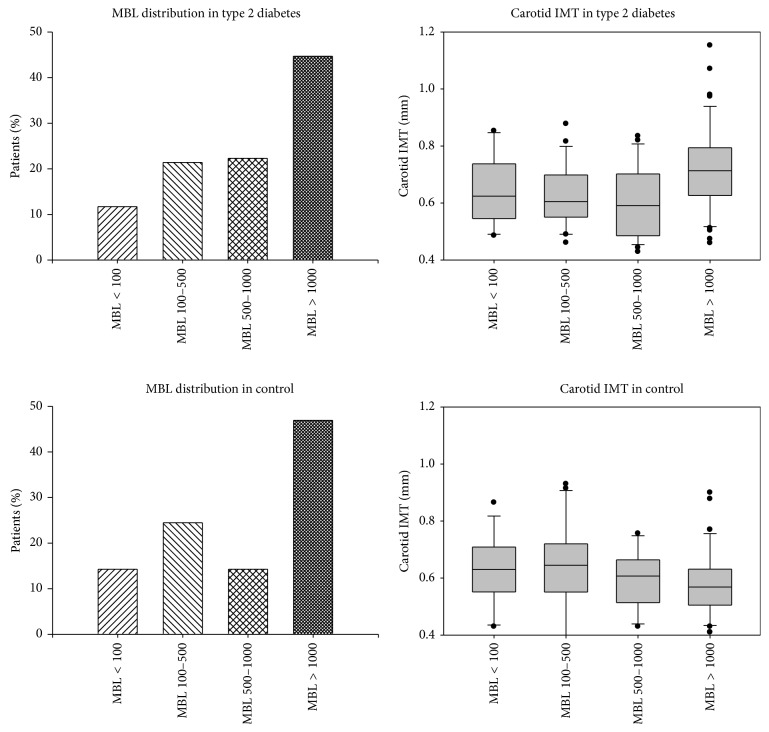
Box and Whisker plots depicting MBL distribution according to various subgroups in type 2 diabetics and control group. Absolute MBL deficiency (<100 ng/mL), intermediate MBL deficiency (100–500 ng/mL), normal MBL (500–1000 ng/mL), and high MBL levels (>1000 ng/mL) subgroups are depicted.

**Figure 3:**
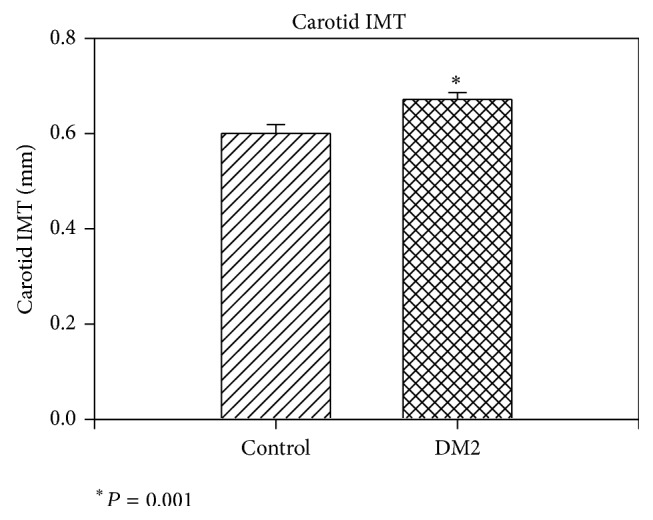
Carotid intima-media thickness (IMT) (mm) in type 2 diabetic patients and controls. IMT was significantly higher in T2DM patients (0.672 ± 0.148) than in controls (0.602 ± 0.128) (*P* = 0.001).

**Figure 4:**
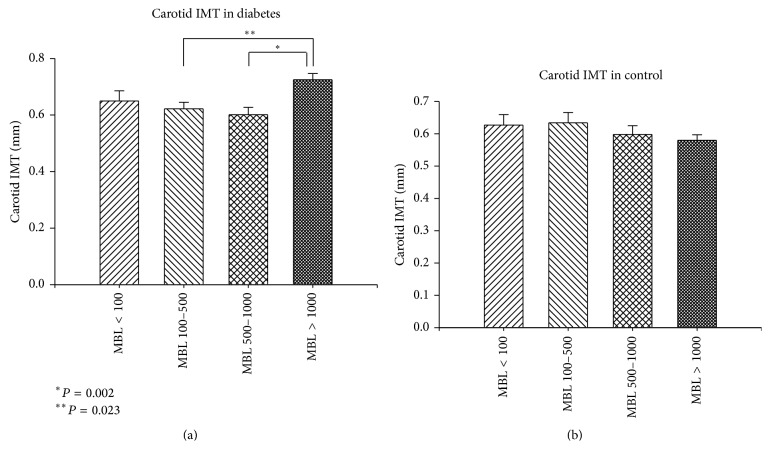
Carotid intima-media thickness (IMT) (mm) in various MBL subgroups in diabetics and controls. Amongst the controls, there was no significant difference in IMT in the four MBL subgroups. In diabetics, lowest IMT was seen in subgroup with normal MBL level (500–1000 ng/mL) while IMT continuously increased with both high MBL and absolute MBL deficiency.

Because of dual role of MBL in determination of CVD risk, subgroups of subjects with absolute functional deficiency (MBL < 100 ng/mL), intermediate deficiency (MBL 100–500 ng/mL), normal MBL (500–1000 ng/mL), and high MBL level (>1000 ng/mL) within both DM2 and control group were analyzed separately. The percentage of patients among four MBL subgroups did not differ in T2DM and control group, as seen in Figure 2. The IMT values of T2DM and control patients in MBL subgroups are also depicted in Figure 2. As expected, IMT was significantly higher in T2DM patients (0.672 ± 0.148) than in controls (0.602 ± 0.128) (*P* = 0.001) (Figure 3). Amongst the controls, there was no significant difference in IMT in the four MBL subgroups. Meanwhile, in T2DM, the lowest IMT was seen in subgroup with normal MBL level (500–1000 ng/mL) while IMT continuously increased with both high MBL and absolute MBL deficiency states (Figure 4). This was especially significant in high MBL T2DM group (0.725 ± 0.148) versus normal MBL T2DM group (0.601 ± 0.122) (*P* = 0.002). Similarly, in high MBL subgroup, IMT in T2DM patients was higher than in controls (0.725 ± 0.148 and 0.58 ± 0.118, resp., *P* < 0.001) (Figure 4).

### 3.2. Correlation between MBL, IMT, and Clinical Parameters

In the T2DM group, among patients with MBL levels above 500, there was a significant correlation between MBL level and IMT (*r* = 0.379, *P* = 0.001). Amongst the diabetics, correlation of IMT and CVD risk factors showed strongest association with age (*r* = 0.537), followed by MBL (*r* = 0.379) and with duration of diabetes (*r* = 0.292). Moreover, ApoA showed a negative correlation with IMT (*r* = −0.325, *P* = 0.03). In controls, the male gender (*P* = 0.005), age (0.024), BMI (0.005), waist circumference (*P* = 0.011), glucose (*P* = 0.009), and HbA1C (*P* = 0.017) were correlated significantly with IMT.

### 3.3. Regression Analysis

Binary logistic regression analysis in high IMT subgroup (>0.7) showed a significant contention with ApoA (*P* = 0.03) and near significant values for age (*P* = 0.053), triglyceride (*P* = 0.053), and cholesterol (*P* = 0.056).

On the basis of backward stepwise multiple regression analysis in T2DM group, the main predictors of IMT are the age (*P* < 0.003), ApoA level (*P* = 0.023), and the MBL (*P* = 0.036), while the total cholesterol level (*P* = 0.074) and HDL (*P* = 0.055) showed near significant prediction. Other clinical factors (ApoB100, ApoB100/ApoA, triglyceride, and LDL) were excluded. In control group, in the backward stepwise multiple regression analysis the male gender (*P* < 0.0001), the HDL level (*P* = 0.098), and ApoB100/ApoA (*P* = 0.0001) were the predictors, while other clinical parameters (cholesterol, HbA1C, ApoB100, TG, glucose, CRP, BMI, waist circumference, MBL, LDL, and ApoA) were excluded (Table 2).

## 4. Discussion

Our results show that IMT continuously increased with both high MBL and absolute MBL deficiency states in T2DM group. This study supports the hypothesis of dual association between MBL levels and IMT in type 2 diabetic patients. It shows for the first time that both high MBL levels and absolute MBL deficiency states may contribute to increases in cIMT in diabetics, as previously demonstrated, both experimentally [26] and in patients with rheumatoid arthritis [24].

*MBL*, an acute phase reactant, is synthesized by the hepatocytes. On binding to specific carbohydrate molecules it activates the complement through mannose associated serine proteases [5, 27]. Although MBL concentration varies, variations within an individual are very small compared to the interindividual differences in a population [4]. In Caucasians, the median concentration is 800–1,000 *μ*g/L (ng/mL) [3]. We used a double monoclonal antibody assay, which is sensitive, and a reproducible method to determine the MBL antigen levels in the sera. It accurately indicates the function estimated by mannan-binding assay or complement activation in the C4b deposition assay. Mutation homozygotes or compound heterozygotes of MBL2 genotypes have profoundly reduced MBL levels. However, due to additional effects of noncoding polymorphisms and some unknown factors, the individual MBL values vary substantially in wild-type homozygotes and mutation heterozygotes, thereby only providing a rough guide to serum MBL concentrations. Since serum MBL levels vary markedly even amongst individuals with identical genotypes, measuring MBL concentration in serum is more reliable than genotyping [14, 20, 28].

Several mechanisms may underlie the atherogenic effect of MBL deficiency. MBL has diverse functions like modulation of inflammation, recognition of altered self-structures, apoptotic cell clearance [29, 30], and removal of antigen-antibody complexes [31, 32]. Thus, in a state of MBL deficiency, apoptotic/damaged cells are not sequestered causing lipid accumulation and atherosclerosis. Furthermore, variant alleles cause defective complement activation due to decreased oligomerization and dysfunctional association with ligands [33], thereby causing impaired clearance of large triglyceride rich very low density lipoproteins (VLDL) [34, 35] and promoting atherosclerosis. Low MBL may lead to enhanced proinflammatory cytokines, such as IL-6 [36] and TNF-alpha [37] which are predictors of CVD.

Furthermore, MBL is an important component of* innate immunity*. Thus, low MBL levels and variant alleles increase infection susceptibility [31]. Acute infections in children have been shown to derange lipid levels and increase IMT [38]. Chronic infection may lead to increased production of serum amyloid A and other acute phase proteins that change the role of HDL from being anti-inflammatory to a proinflammatory state [39]. Several studies have shown an association between CVD and various infectious agents including cytomegalovirus,* Helicobacter pylori,* and* Chlamydia pneumoniae (C. pneumoniae)* [40].* C. pneumoniae* infection provokes severe CAD in individuals with variant alleles (low MBL) [41].

In heart ischemia reperfusion model, inhibition of lectin pathway significantly decreases infarct size in type 2 diabetic rats [42], whereas MBL plays a critical role in type 1 diabetic mice [43]. Diabetes may cause advanced glycation end products of endothelial surfaces causing increased MBL deposition with subsequent complement activation, tissue injury, and atherosclerosis [44]. Terminal complement deposits (C5b-9) were found in the intima of atherosclerotic lesions [45]. Hyperglycemia mediates O-glycation of N-acetylglucosamine (GlcNAc) [46] of various proteins including LDL, membrane phospholipids, and apolipoprotein B [44]. MBL strongly binds to these GlcNAc residues [47] causing increased susceptibility to oxidation and leads to functional alterations in LDL clearance [31]. Moreover, glycation causes inactivation of CD59, a regulatory protein involved in decreasing endothelial susceptibility to membrane attack complex mediated injury [48]. The above mechanisms could act towards promoting atherosclerosis in T2D with high serum MBL.

We hypothesize that this dual role of MBL with IMT in T2DM can be explained in view of various functions of MBL. Absolute MBL deficiency may promote atherogenesis by enhancing VLDL, proinflammatory cytokines, and predisposing to chronic infections which alters functions of HDL. It may also restrict clearing of early atherosclerotic lesions due to dysfunctional MBL oligomerization. Furthermore, combination of high MBL level and hyperglycemia associated with advanced glycation products, fructosamine, altered LDL clearance, and defective CD59 may increase atherosclerosis.

In conclusion, the data presented demonstrate for the first time the potential dual role of MBL in pathologic processes leading to atherosclerosis in diabetic patients. MBL deficiency and excess MBL are a risk factor for subclinical carotid artery atherosclerosis in T2DM. In addition to the potential role of MBL in the atherogenesis it may also be used as a marker of macrovascular disease, as both low and high levels may indicate the susceptibility for atherosclerosis in T2DM. Early screening of patients may help distinguish high risk group and guide prophylactic initiatives. MBL can be assessed as a member of inherited nontraditional risk factors influencing the development of atherosclerosis significantly in diabetic patients.

However, the main limitation of this study is the low number of patients and therefore further studies with larger population size are required to ascertain the exact role of MBL in atherosclerosis.

## Figures and Tables

**Table 1:** Anthropometric and laboratory data of type 2 diabetics (T2DM) and control group of our study population.

Parameter	T2DM group	Control group	Significance
	*n* = 103	*n* = 98	
Age (year)	49.78 ± 0.81	48.66 ± 1.29	*P* = 0.929
Duration of diabetes (years)	8.64 ± 0.62		
Gender (M/F)	65/38	52/46	*P* = 0.151
BMI (kg/m^2^)	31.68 ± 0.51	29.67 ± 1.0	*P* = 0.299
Waist circumference (cm)	110.74 ± 1.33	104.2 ± 2.6	*P* = 0.028
Smoking (Y/N)	22/81	23/75	*P* = 0.423
Hypertension (Y/N)	62/41	30/68	*P* < 0.001
Glucose (mmol/L)	10.17 ± 0.43	5.3 ± 0.13	*P* < 0.001
HbA1C (%)	8.18 ± 0.17	5.59 ± 0.07	*P* < 0.001
CRP (mg/L)	5.8 ± 1.08	5.63 ± 0.9	*P* = 0.98
Triglyceride (mmol/L)	2.87 ± 0.29	1.53 ± 0.11	*P* = 0.001
Cholesterol (mmol/L)	5.03 ± 0.13	5.37 ± 0.11	*P* = 0.045
HDL-cholesterol (mmol/L)	1.19 ± 0.03	2.29 ± 0.1	*P* < 0.001
LDL-cholesterol (mmol/L)	2.91 ± 0.09	2.28 ± 0.12	*P* < 0.001
ApoA (g/L)	1.41 ± 0.03	1.56 ± 0.03	*P* = 0.001
ApoB100 (g/L)	0.91 ± 0.03	0.96 ± 0.03	*P* = 0.214
Lp(a) (mg/L)	344.9 ± 50	299.1 ± 39.1	*P* = 0.704

**Table 2:** Results of backward stepwise multiple regression analysis with beta coefficient and significance. Age, ApoA, and MBL were significantly associated with carotid intima-media thickness.

Parameter	Beta coefficient	Significance
Age (year)	0.400	*P* = 0.003
ApoA (g/L)	−0.627	*P* = 0.023
MBL	0.272	*P* = 0.036

Cholesterol (mmol/L)	0.253	*P* = 0.074
HDL-cholesterol (mmol/L)	0.492	*P* = 0.055

ApoB100 (g/L)	0.021	*P* = 0.952
BMI	0.096	*P* = 0.537
ApoB100/ApoA	0.135	*P* = 0.68
Triglyceride (mmol/L)	0.035	*P* = 0.841
LDL-cholesterol (mmol/L)	0.107	*P* = 0.647

## References

[1] Stamler J., Vaccaro O., Neaton J. D., Wentworth D. (1993). Diabetes, other risk factors, and 12-yr cardiovascular mortality for men screened in the multiple risk factor intervention trial. *Diabetes Care*.

[2] Ray A., Huisman M. V., Tamsma J. T. (2009). The role of inflammation on atherosclerosis, intermediate and clinical cardiovascular endpoints in type 2 diabetes mellitus. *European Journal of Internal Medicine*.

[3] Hansen T. K., Tarnow L., Thiel S. (2004). Association between mannose-binding lectin and vascular complications in type 1 diabetes. *Diabetes*.

[4] Hansen T. K., Thiel S., Knudsen S. T. (2003). Elevated levels of mannan-binding lectin in patients with type 1 diabetes. *Journal of Clinical Endocrinology & Metabolism*.

[5] Nuytinck L., Shapiro F. (2004). Mannose-binding lectin: laying the stepping stones from clinical research to personalized medicine. *Personalized Medicine*.

[6] Thiel S., Holmskov U., Hviid L., Laursen S. B., Jensenius J. C. (1992). The concentration of the C-type lectin, mannan-binding protein, in human plasma increases during an acute phase response. *Clinical and Experimental Immunology*.

[7] Madsen H. O., Videm V., Svejgaard A., Svennevig J. L., Garred P. (1998). Association of mannose-binding-lectin deficiency with severe atherosclerosis. *The Lancet*.

[8] Rugonfalvi-Kiss S., Endrész V., Madsen H. O. (2002). Association of *Chlamydia pneumoniae* with coronary artery disease and its progression is dependent on the modifying effect of mannose-binding lectin. *Circulation*.

[9] Øhlenschlæger T., Garred P., Madsen H. O., Jacobsen S. (2004). Mannose-binding lectin variant alleles and the risk of arterial thrombosis in systemic lupus erythematosus. *The New England Journal of Medicine*.

[10] Best L. G., Davidson M., North K. E. (2004). Prospective analysis of mannose-binding lectin genotypes and coronary artery disease in American Indians: the Strong Heart Study. *Circulation*.

[11] Biezeveld M. H., Kuipers I. M., Geissler J. (2003). Association of mannose-binding lectin genotype with cardiovascular abnormalities in Kawasaki disease. *The Lancet*.

[12] Limnell V., Aittoniemi J., Vaarala O. (2002). Association of mannan-binding lectin deficiency with venous bypass graft occlusions in patients with coronary heart disease. *Cardiology*.

[13] Hegele R. A., Ban M. R., Anderson C. M., Spence J. D. (2000). Infection-susceptibility alleles of mannose-binding lectin are associated with increased carotid plaque area. *Journal of Investigative Medicine*.

[14] Keller T. T., Van Leuven S. I., Meuwese M. C. (2006). Serum levels of mannose-binding lectin and the risk of future coronary artery disease in apparently healthy men and women. *Arteriosclerosis, Thrombosis, and Vascular Biology*.

[15] Rugonfalvi-Kiss S., Dósa E., Madsen H. O. (2005). High rate of early restenosis after carotid eversion endarterectomy in homozygous carriers of the normal mannose-binding lectin genotype. *Stroke*.

[16] Siezenga M. A., Chandie Shaw P. K., Daha M. R., Rabelink T. J., Berger S. P. (2011). Low Mannose-Binding Lectin (MBL) genotype is associated with future cardiovascular events in type 2 diabetic South Asians. A prospective cohort study. *Cardiovascular Diabetology*.

[17] Mellbin L. G., Hamsten A., Malmberg K. (2010). Mannose-binding lectin genotype and phenotype in patients with type 2 diabetes and myocardial infarction: a report from the DIGAMI 2 trial. *Diabetes Care*.

[18] Hansen T. K., Gall M.-A., Tarnow L. (2006). Mannose-binding lectin and mortality in type 2 diabetes. *Archives of Internal Medicine*.

[19] Elawa G., Aoudallah A. M., Hasaneen A. E., El-Hammady A. M. (2011). The predictive value of serum mannan-binding lectin levels for diabetic control and renal complications in type 2 diabetic patients. *Saudi Medical Journal*.

[20] Saevarsdottir S., Oskarsson O. O., Aspelund T. (2005). Mannan binding lectin as an adjunct to risk assessment for myocardial infarction in individuals with enhanced risk. *The Journal of Experimental Medicine*.

[21] Järvisalo M. J., Raitakari M., Toikka J. O. (2004). Endothelial dysfunction and increased arterial intima-media thickness in children with type 1 diabetes. *Circulation*.

[22] El-Sherif W. T., Herdan O. M., Osman M. H., Alkady E. A. M. (2010). Mannose binding lectin gene polymorphism and preclinical carotid atherosclerosis in patients with systemic lupus erythematosus. *The Egyptian Journal of immunology/Egyptian Association of Immunologists*.

[23] Troelsen L. N., Garred P., Christiansen B., Torp-Pedersen C., Jacobsen S. (2010). Genetically determined serum levels of mannose-binding lectin correlate negatively with common carotid intima-media thickness in systemic lupus erythematosus. *The Journal of Rheumatology*.

[24] Troelsen L. N., Garred P., Christiansen B. (2010). Double role of mannose-binding lectin in relation to carotid intima-media thickness in patients with rheumatoid arthritis. *Molecular Immunology*.

[25] Papp M., Lakatos P. L., Harsfalvi J. (2010). Mannose-binding lectin level and deficiency is not associated with inflammatory bowel diseases, disease phenotype, serology profile, and NOD2/CARD15 genotype in a large Hungarian cohort. *Human Immunology*.

[26] Matthijsen R. A., de Winther M. P. J., Kuipers D. (2009). Macrophage-specific expression of mannose-binding lectin controls atherosclerosis in low-density lipoprotein receptor–deficient mice. *Circulation*.

[27] Wallis R. (2007). Interactions between mannose-binding lectin and MASPs during complement activation by the lectin pathway. *Immunobiology*.

[28] Steffensen R., Thiel S., Varming K., Jersild C., Jensenius J. C. (2000). Detection of structural gene mutations and promoter polymorphisms in the mannan-binding lectin (MBL) gene by polymerase chain reaction with sequence-specific primers. *Journal of Immunological Methods*.

[29] Nauta A. J., Raashou-Jensen N., Roos A. (2003). Mannose-binding lectin engagement with late apoptotic and necrotic cells. *European Journal of Immunology*.

[30] Ogden C. A., deCathelineau A., Hoffmann P. R. (2001). C1q and mannose binding lectin engagement of cell surface calreticulin and CD91 initiates macropinocytosis and uptake of apoptotic cells. *The Journal of Experimental Medicine*.

[31] Dommett R. M., Klein N., Turner M. W. (2006). Mannose-binding lectin in innate immunity: past, present and future. *Tissue Antigens*.

[32] Roos A., Bouwman L. H., van Gijlswijk-Janssen D. J., Faber-Krol M. C., Stahl G. L., Daha M. R. (2001). Human IgA activates the complement system via the Mannan-Binding lectin pathway. *The Journal of Immunology*.

[33] Garred P., Larsen F., Madsen H. O., Koch C. (2003). Mannose-binding lectin deficiency—revisited. *Molecular Immunology*.

[34] Alipour A., van Oostrom A. J., van Wijk J. P. (2007). Abstract 1098: deficiency of mannose binding lectin is associated with increased postprandial VLDL1 despite normal fasting plasma triglycerides. *Circulation*.

[35] Alipour A., van Oostrom A. J. H. H. M., van Wijk J. P. H. (2009). Mannose binding lectin deficiency and triglyceride-rich lipoprotein metabolism in normolipidemic subjects. *Atherosclerosis*.

[36] Tzoulaki I., Murray G. D., Lee A. J., Rumley A., Lowe G. D. O., Fowkes F. G. R. (2005). C-reactive protein, interleukin-6, and soluble adhesion molecules as predictors of progressive peripheral atherosclerosis in the general population: edinburgh artery study. *Circulation*.

[37] Ridker P. M., Rifai N., Pfeffer M., Sacks F., Lepage S., Braunwald E. (2000). Elevation of tumor necrosis factor-*α* and increased risk of recurrent coronary events after myocardial infarction. *Circulation*.

[38] Liuba P., Persson J., Luoma J., Ylä-Herttuala S., Pesonen E. (2003). Acute infections in children are accompanied by oxidative modification of LDL and decrease of HDL cholesterol, and are followed by thickening of carotid intima-media. *European Heart Journal*.

[39] van Lenten B. J., Hama S. Y., de Beer F. C. (1995). Anti-inflammatory HDL becomes pro-inflammatory during the acute phase response: loss of protective effect of HDL against LDL oxidation in aortic wall cell cocultures. *The Journal of Clinical Investigation*.

[40] Libby P., Egan D., Skarlatos S. (1997). Roles of infectious agents in atherosclerosis and restenosis: an assessment of the evidence and need for future research. *Circulation*.

[41] Rugonfalvi-Kiss S., Endrész V., Madsen H. O. (2002). Association of *Chlamydia pneumoniae* with coronary artery disease and its progression is dependent on the modifying effect of mannose-binding lectin. *Circulation*.

[42] La Bonte L. R., Dokken B., Davis-Gorman G., Stahl G. L., McDonagh P. F. (2009). The mannose-binding lectin pathway is a significant contributor to reperfusion injury in the type 2 diabetic heart. *Diabetes and Vascular Disease Research*.

[43] Busche M. N., Walsh M. C., McMullen M. E., Guikema B. J., Stahl G. L. (2008). Mannose-binding lectin plays a critical role in myocardial ischaemia and reperfusion injury in a mouse model of diabetes. *Diabetologia*.

[44] Aronson D., Rayfield E. J. (2002). How hyperglycemia promotes atherosclerosis: molecular mechanisms. *Cardiovascular Diabetology*.

[45] Vlaicu R., Niculescu F., Rus H. G., Cristea A. (1985). Immunohistochemical localization of the terminal C5b-9 complement complex in human aortic fibrous plaque. *Atherosclerosis*.

[46] Hart G. W. (1997). Dynamic O-linked glycosylation of nuclear and cytoskeletal proteins. *Annual Review of Biochemistry*.

[47] Royle L., Roos A., Harvey D. J. (2003). Secretory IgA *N*- and *O*-glycans provide a link between the innate and adaptive immune systems. *The Journal of Biological Chemistry*.

[48] Acosta J., Hettinga J., Flückiger R. (2000). Molecular basis for a link between complement and the vascular complications of diabetes. *Proceedings of the National Academy of Sciences of the United States of America*.

